# Stagnation-point flow over a stretching/shrinking sheet in a nanofluid

**DOI:** 10.1186/1556-276X-6-623

**Published:** 2011-12-08

**Authors:** Norfifah Bachok, Anuar Ishak, Ioan Pop

**Affiliations:** 1 Department of Mathematics and Institute for Mathematical Research, Universiti Putra Malaysia, 43400 UPM Serdang, Selangor, Malaysia; 2 School of Mathematical Sciences, Faculty of Science and Technology, Universiti Kebangsaan Malaysia, 43600 UKM Bangi, Selangor, Malaysia; 3 Faculty of Mathematics, University of Cluj, CP 253, 3400 Cluj, Romania

**Keywords:** nanofluids, stagnation-point flow, heat transfer, stretching/shrinking sheet, dual solutions.

## Abstract

An analysis is carried out to study the steady two-dimensional stagnation-point flow of a nanofluid over a stretching/shrinking sheet in its own plane. The stretching/shrinking velocity and the ambient fluid velocity are assumed to vary linearly with the distance from the stagnation point. The similarity equations are solved numerically for three types of nanoparticles, namely copper, alumina, and titania in the water-based fluid with Prandtl number Pr = 6.2. The skin friction coefficient, Nusselt number, and the velocity and temperature profiles are presented graphically and discussed. Effects of the solid volume fraction φ on the fluid flow and heat transfer characteristics are thoroughly examined. Different from a stretching sheet, it is found that the solutions for a shrinking sheet are non-unique.

## Introduction

Stagnation-point flow, describing the fluid motion near the stagnation region of a solid surface exists in both cases of a fixed or moving body in a fluid. The two-dimensional stagnation-point flow towards a stationary semi-infinite wall was first studied by Hiemenz [1], who used a similarity transformation to reduce the Navier-Stokes equations to nonlinear ordinary differential equations. This problem has been extended by Homann [2] to the case of axisymmetric stagnation-point flow. The combination of both stagnation-point flows past a stretching surface was considered by Mahapatra and Gupta [3,4]. There are two conditions that the flow towards a shrinking sheet is likely to exist, whether an adequate suction on the boundary is imposed [5] or a stagnation flow is considered [6]. Wang [6] investigated both two-dimensional and axisymmetric stagnation flow towards a shrinking sheet in a viscous fluid. He found that solutions do not exist for larger shrinking rates and non-unique in the two-dimensional case. After this pioneering work, the flow field over a stagnation point towards a stretching/shrinking sheet has drawn considerable attention and a good amount of literature has been generated on this problem [7-10].

All studies mentioned above refer to the stagnation-point flow towards a stretching/shrinking sheet in a viscous and Newtonian fluid. The present paper deals with the problem of a steady boundary-layer flow, heat transfer, and nanoparticle fraction over a stagnation point towards a stretching/shrinking sheet in a nanofluid, with water as the based fluid. Most conventional heat transfer fluids, such as water, ethylene glycol, and engine oil, have limited capabilities in terms of thermal properties, which, in turn, may impose serve restrictions in many thermal applications. On the other hand, most solids, in particular, metals, have thermal conductivities much higher, say, by one to three orders of magnitude, compared with that of liquids. Hence, one can then expect that fluid-containing solid particles may significantly increase its conductivity. The flow over a continuously stretching surface is an important problem in many engineering processes with applications in industries such as the hot rolling, wire drawing, paper production, glass blowing, plastic films drawing, and glass-fiber production. The quality of the final product depends on the rate of heat transfer at the stretching surface. On the other hand, the new type of shrinking sheet flow is essentially a backward flow as discussed by Goldstein [11] and it shows physical phenomena quite distinct from the forward stretching flow [12]. The enhanced thermal behavior of nanofluids could provide a basis for an enormous innovation for heat transfer intensification for the processes and applications mentioned above.

Many of the publications on nanofluids are about understanding of their behaviors so that they can be utilized where straight heat transfer enhancement is paramount as in many industrial applications, nuclear reactors, transportation, electronics as well as biomedicine and food. The broad range of current and future applications involving nanofluids have been given by Wong and Leon [13]. Nanofluid as a smart fluid, where heat transfer can be reduced or enhanced at will, has also been reported. These fluids enhance thermal conductivity of the base fluid enormously, which is beyond the explanation of any existing theory. They are also very stable and have no additional problems, such as sedimentation, erosion, additional pressure drop and non-Newtonian behavior, due to the tiny size of nanoelements and the low volume fraction of nanoelements required for conductivity enhancement. These suspended nanoparticles can change the transport and thermal properties of the base fluid. The comprehensive references on nanofluids can be found in the recent book by Das et al. [14] and in the review papers by Buongiorno [15], Daungthongsuk and Wongwises [16], Trisaksri and Wongwises [17], Ding et al. [18], Wang and Mujumdar [19-21], Murshed et al. [22], and Kakaç and Pramuanjaroenkij [23].

The nanofluid model proposed by Buongiorno [15] was very recently used by Nield and Kuznetsov [24,25], Kuznetsov and Neild [26,27], Khan and Pop [28], and Bachok et al. [29] in their papers. The paper by Khan and Pop [28] is the first which considered the problem on stretching sheet in nanofluids. Different from the above model, the present paper considers a problem using the nanofluid model proposed by Tiwari and Das [30], which was also used by several authors (cf. Abu-Nada [31], Muthtamilselvan et al. [32], Abu-Nada and Oztop [33], Talebi et al. [34], Ahmad et al. [35], Bachok et al. [36,37], Yacob et al. [38]). The model proposed by Buongiorno [15] studies the Brownian motion and the thermophoresis on the heat transfer characteristics, while the model by Tiwari and Das [30] analyzes the behavior of nanofluids taking into account the solid volume fraction. In the present paper, we analyze the effects of the solid volume fraction and the type of the nanoparticles on the fluid flow and heat transfer characteristics of a nanofluid over a stretching/shrinking sheet.

## Mathematical formulation

**Figure 1:**
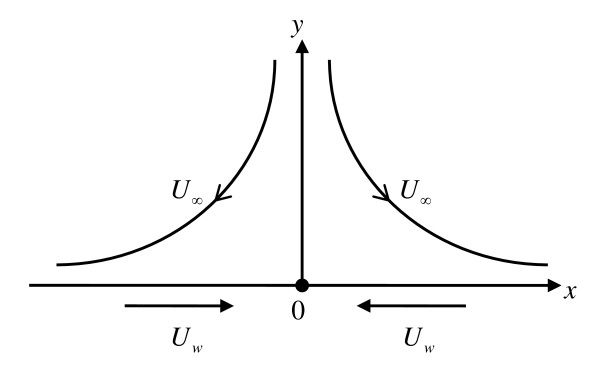
**Physical model and coordinate system**.

Consider the flow of an incompressible nanofluid in the region *y *> 0 driven by a stretching/shrinking surface located at *y *= 0 with a fixed stagnation point at *x *= 0 as shown in Figure 1. The stretching/shrinking velocity *U*_w _(*x*) and the ambient fluid velocity *U*_∞ _(*x*) are assumed to vary linearly from the stagnation point, i.e., *U*_w _(*x*) = *ax *and *U*_∞ _(*x*) = *bx*, where *a *and *b *are constant with *b *> 0. We note that *a *> 0 and *a *< 0 correspond to stretching and shrinking sheets, respectively. The simplified two-dimensional equations governing the flow in the boundary layer of a steady, laminar, and incompressible nanofluid are (see [35])

(1) $$\frac{\partial u}{\partial x}+\frac{\partial v}{\partial y}=0,$$

(2) $$u\frac{\partial u}{\partial x}+v\frac{\partial u}{\partial y}=U_{\infty }\frac{dU_{\infty }}{dx}+\frac{\mu_{\text{nf}}}{\rho_{\text{nf}}}\frac{\partial^{2}u}{\partial y^{2}},$$

and

(3) $$u\frac{\partial T}{\partial x}+v\frac{\partial T}{\partial y}=\alpha_{\text{nf}}\frac{\partial^{2}T}{\partial y^{2}}$$

subject to the boundary conditions

(4) $$\begin{array}{c} u=U_{\text{w}}\left(x\right),\;v=0,\;T=T_{\text{w}}\;\text{at}\;y=0,\\ u\to U_{\infty }\left(x\right),\;T\to T_{\infty }\;\text{as}\;y\to \infty , \end{array}$$

where *u *and *v *are the velocity components along the *x*- and *y*- axes, respectively, *T *is the temperature of the nanofluid, *μ*_nf _is the viscosity of the nanofluid, *α*_nf _is the thermal diffusivity of the nanofluid and *ρ*_nf _is the density of the nanofluid, which are given by Oztop and Abu-Nada [39]

(5) $$\begin{array}{c} \alpha_{\text{nf}}=\frac{k_{\text{nf}}}{{\left(\rho C_{p}\right)}_{\text{nf}}},\;\;\;\;\;\rho_{\text{nf}}=\left(1-\varphi \right)\rho_{\text{f}}+\varphi \rho_{\text{s}},\;\;\;\mu_{\text{nf}}=\;\frac{\mu_{\text{f}}}{{\left(1-\varphi \right)}^{2.5}},\\ {\left(\rho C_{p}\right)}_{\text{nf}}=\left(1-\varphi \right){\left(\rho C_{p}\right)}_{\text{f}}+\varphi {\left(\rho C_{p}\right)}_{\text{s}},\;\;\frac{k_{\text{nf}}}{k_{\text{f}}}=\frac{\left(k_{\text{s}}+2k_{\text{f}}\right)-2\varphi \left(k_{\text{f}}-k_{\text{s}}\right)}{\left(k_{\text{s}}+2k_{\text{f}}\right)+\varphi \left(k_{\text{f}}-k_{\text{s}}\right)} \end{array}$$

Here, φ is the nanoparticle volume fraction, (*ρ*C_*p*_)_nf _is the heat capacity of the nanofluid, *k*_nf _is the thermal conductivity of the nanofluid, *k*_f _and *k*_s _are the thermal conductivities of the fluid and of the solid fractions, respectively, and *ρ*_f _and *ρ*_s _are the densities of the fluid and of the solid fractions, respectively. It should be mentioned that the use of the above expression for *k*_nf _is restricted to spherical nanoparticles where it does not account for other shapes of nanoparticles [31]. Also, the viscosity of the nanofluid *μ*_nf _has been approximated by Brinkman [40] as viscosity of a base fluid *μ*_f _containing dilute suspension of fine spherical particles.

The governing Eqs. 1, 2, and 3 subject to the boundary conditions (4) can be expressed in a simpler form by introducing the following transformation:

(6) $$\eta ={\left(\frac{b}{\nu_{\text{f}}}\right)}^{1∕2}y,\;\;\psi ={\left(\nu_{\text{f}}b\right)}^{1∕2}x\;f\left(\eta \right),\;\theta \left(\eta \right)=\frac{T-T_{\infty }}{T_{\text{w}}-T_{\infty }}$$

where *η *is the similarity variable and *ψ *is the stream function defined as *u *= ∂*ψ/∂y *and *v *= -∂*ψ/∂x*, which identically satisfies Eq. 1. Employing the similarity variables (6), Eqs. 2 and 3 reduce to the following ordinary differential equations:

(7) $$\frac{1}{{\left(1-\varphi \right)}^{2.5}\left(1-\varphi +\varphi \rho_{\text{s}}∕\rho_{\text{f}}\right)}f^{\prime \prime \prime }+ff^{\prime \prime }-{f{}^{\prime }}^{2}+1=0$$

(8) $$\frac{1}{Pr}\frac{k_{\text{nf}}∕k_{\text{f}}}{\left[1-\varphi +\varphi {\left(\rho C_{p}\right)}_{\text{s}}∕{\left(\rho C_{p}\right)}_{\text{f}}\right]}\theta^{\prime \prime }+f\theta^{\prime }=0$$

subjected to the boundary conditions (4) which become

(9) $$\begin{array}{c} f\left(0\right)=0,\;\;f^{\prime }\left(0\right)=\varepsilon ,\;\;\theta \left(0\right)=1\\ f^{\prime }\left(\eta \right)\to 1,\;\;\theta \left(\eta \right)\to 0\;\text{as}\;\eta \to \infty . \end{array}$$

In the above equations, primes denote differentiation with respect to *η*, *Pr*(= *v*_f_/*α*_f_) is the Prandtl number, and *ε *is the velocity ratio parameter defined as

(10) $$\varepsilon =\frac{a}{b}$$

where *ε *> 0 for stretching and *ε *< 0 for shrinking.

The physical quantities of interest are the skin friction coefficient *C*_f _and the local Nusselt number Nu_*x*_, which are defined as

(11) $$C_{\text{f}}=\frac{\tau_{\text{w}}}{\rho_{\text{f}}U_{\infty }^{2}},\;\text{N}{\text{u}}_{x}=\frac{xq_{{}_{\text{w}}}}{k_{\text{f}}\left(T_{\text{w}}-T_{\infty }\right)},$$

where the surface shear stress *τ*_w _and the surface heat flux *q*_w _are given by

(12) $$\tau_{\text{w}}=\mu_{\text{nf}}{\left(\frac{\partial u}{\partial y}\right)}_{y=0},\;q_{\text{w}}=-k_{\text{nf}}{\left(\frac{\partial T}{\partial y}\right)}_{y=0},$$

with *μ*_nf _and *k*_nf _being the dynamic viscosity and thermal conductivity of the nanofluids, respectively. Using the similarity variables (6), we obtain

(13) $$C_{\text{f}}Re_{x}^{1∕2}=\frac{1}{{\left(1-\varphi \right)}^{2.5}}f^{\prime \prime }\left(0\right),$$

(14) $$\text{N}{\text{u}}_{x}∕{\text{Re}}_{x}^{1∕2}=-\frac{k_{\text{nf}}}{k_{\text{f}}}\theta^{\prime }\left(0\right),$$

where Re_x _= *U*_∞_*x */*ν*_f _is the local Reynolds number.

## Results and discussion

Numerical solutions to the governing ordinary differential Eqs. 7 and 8 with the boundary conditions (9) were obtained using a shooting method. The dual solutions were obtained by setting different initial guesses for the missing values of *f"*(0) and *θ'*(0), where all profiles satisfy the boundary conditions (9) asymptotically but with different shapes. The effects of the solid volume fraction of nanofluid *φ *and the Prandtl number Pr are analyzed for three different nanofluids, namely copper (Cu)-water, alumina (Al_2_O_3_)-water, and titania (TiO_2_)-water, as the working fluids. Following Oztop and Abu-Nada [39] or Khanafer et al. [41], the value of the Prandtl number Pr is taken as 6.2 (water) and the volume fraction of nanoparticles is from 0 to 0.2 (0 ≤ *φ *≤ 0.2) in which *φ *= 0 corresponds to the regular fluid. The thermophysical properties of the base fluid and the nanoparticles are listed in Table 1. Comparisons with previously reported data available in the literature (for viscous fluid) are made for several values of *ε*, as presented in Table 2, which show a favorable agreement, and thus give confidence that the numerical results obtained are accurate. Moreover, the values of *f"*(0) for *φ *≠ 0 are also included in Table 2 for future references. The numerical values of $C_{\text{f}}{\text{Re}}_{x}^{1/2}$ and $\text{N}{\text{u}}_{x}{\text{Re}}_{x}^{-1/2}$ are presented in Tables 3 and 4, which show a favorable agreement with previous investigation for the case *m *= 1 in Yacob et al. [42]. These tables show that the skin friction and Nusselt number have greater values for Cu than for Al_2_O_3 _and TiO_2_. This is due to the physical properties of fluid and nanoparticles (i.e., thermal conductivity of Cu is much higher than that of Al_2_O_3 _and TiO_2_), see Table 1.

**Table 1:** Thermophysical properties of fluid and nanoparticles [39]

Physical properties	Fluid phase (water)	Cu	**Al**_**2**_**O**_**3**_	**TiO**_**2**_
*C_p_*(J/kg K)	4179	385	765	686.2
*ρ*(kg/m^3^)	997.1	8933	3970	4250
*k*(W/mK)	0.613	400	40	8.9538

**Table 2:** Values of *f*″(0) for some values of *ε *and *φ *for Cu-water working fluid

*ε*	Wang [6]	Present results		
	*φ = *0	*φ = *0	*φ = *0.1	*φ = *0.2
2	-1.88731	-1.887307	-2.217106	-2.298822
1	0	0	0	0
0.5	0.71330	0.713295	0.837940	0.868824
0	1.232588	1.232588	1.447977	1.501346
-0.5	1.49567	1.495670	1.757032	1.821791
-1	1.32882	1.328817	1.561022	1.618557
	[0]	[0]	[0]	[0]
-1.15	1.08223	1.082231	1.271347	1.318205
	[0.116702]	[0.116702]	[0.137095]	[0.142148]
-1.2		0.932473	1.095419	1.135794
		[0.233650]	[0.274479]	[0.284596]
-1.2465	0.55430	0.584281	0.686379	0.711679
		[0.554297]	[0.651161]	[0.675159]

"[ ]" second solution

**Table 3:** Values of CfRex1/2 for some values of *ε *and *φ*

*ε*	*φ*	**Yacob et al. **[42]			Present results		
		Cu-water	Al_2_O_3_-water	TiO_2_-water	Cu-water	Al_2_O_3_-water	TiO_2_-water
-0.5	0.1				2.2865	1.9440	1.9649
	0.2				3.1826	2.4976	2.5413
0	0.1	1.8843	1.6019	1.6192	1.8843	1.6019	1.6192
	0.2	2.6226	2.0584	2.0942	2.6226	2.0584	2.0942
0.5	0.1				1.0904	0.9271	0.9371
	0.2				1.5177	1.1912	1.2118

**Table 4:** Values of NuxRex-1/2 for some values of *ε *and *φ*

*ε*	*φ*	**Yacob et al. **[42]			Present results		
		Cu-water	Al_2_O_3_-water	TiO_2_-water	Cu-water	Al_2_O_3_-water	TiO_2_-water
-0.5	0.1				0.8385	0.7272	0.7082
	0.2				1.0802	0.8878	0.8423
0	0.1	1.4043	1.3305	1.3010	1.4043	1.3305	1.3010
	0.2	1.6692	1.5352	1.4691	1.6692	1.5352	1.4691
0.5	0.1				1.8724	1.8278	1.7898
	0.2				2.1577	2.0700	1.9867

**Figure 2:**
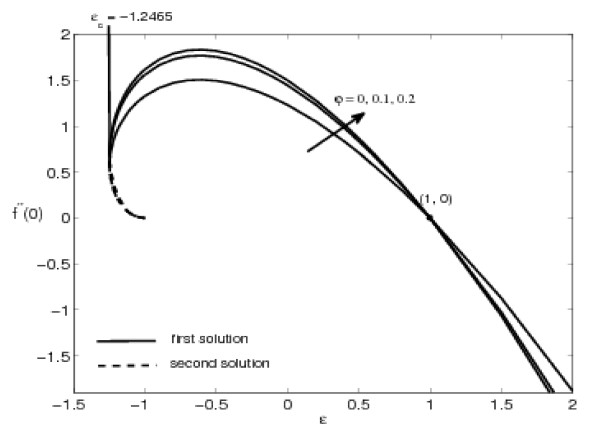
**Variation of *f*"(0) with *ε *for some values of *φ *(0 ≤ *φ *≤ 0.2) for Cu-water working fluid and Pr = 6.2**.

**Figure 3:**
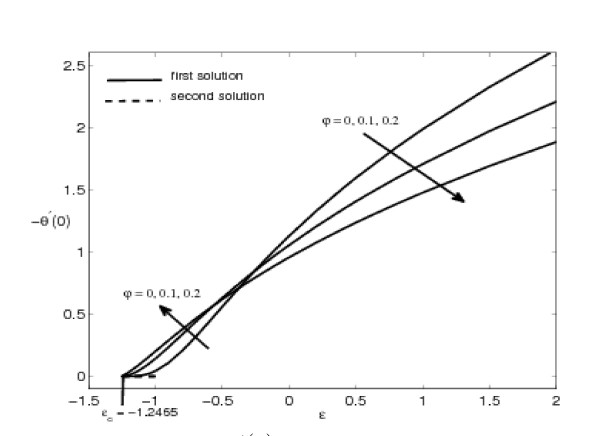
**Variation of -*θ'*(0) with *ε *for some values of *φ *(0 ≤ *φ *≤ 0.2) for Cu-water working fluid and Pr = 6.2**.

**Figure 4:**
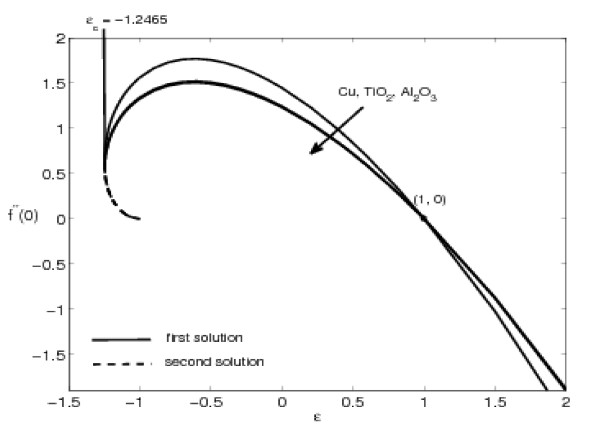
**Variation of *f*"(0) with *ε *for different nanoparticles with *φ *= 0.1 and Pr = 6.2**.

**Figure 5:**
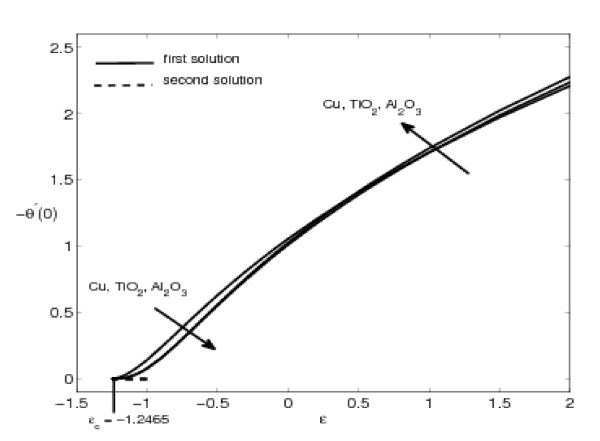
**Variation of -*θ'*(0) with *ε *for different nanoparticles with *φ *= 0.1 and Pr = 6.2**.

The variations of *f"*(0) and -*θ'*(0) with *ε *are shown in Figures 2, 3, 4, and 5 for some values of the velocity ratio parameter *ε *and nanoparticle volume fraction *φ*. These figures show that there are regions of unique solutions for *ε *> -1, dual solutions for *ε_c _*< ε ≤ -1 and no solutions for *ε *<*ε_c _*< 0, where *ε_c _*is the critical value of *ε*. Based on our computation, *ε_c _*= -1.2465. This value of *ε_c _*is in agreement with those reported by Wang [6], Ishak et al. [8] and Bachok et al. [9,10]. Further, it should be mentiond that the first solutions of *f*"(0) and -*θ'*(0) are stable and physically realizable, while the second solutions are not. The procedure for showing this has been described by Weidman et al. [43], Merkin [44], and very recently by Postelnicu and Pop [45], so that we will not repeat it here. The results presented in Figure 2 also indicate that the value of *f*"(0) is zero when *ε *= 1. This is due to the fact that there is no friction at the fluid-solid interface when the fluid and the solid boundary move with the same velocity. The value of *f*"(0) is positive when *ε *< 1 and is negative when *ε *> 1. Physically positive value of *f*"(0) means the fluid exerts a drag force on the solid boundary and negative value means the opposite. We notice that *ε *= 0 correspond to Hiemenz [1] flow, and *ε *= 1 is a degenerate inviscid flow where the stretching matches the conditions at infinity [46].

**Figure 6:**
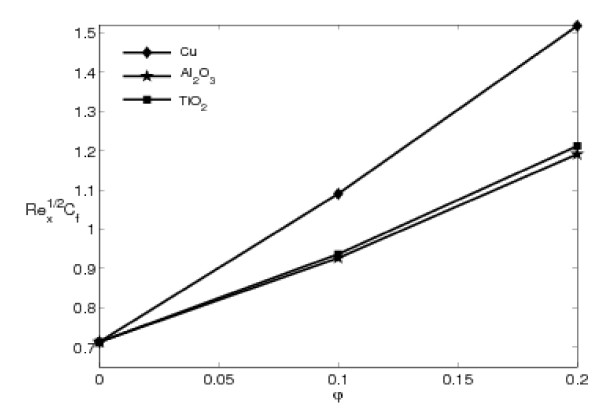
**Variation of the skin friction coefficient CfRex1/2 with *φ *for different nanoparticles with *ε *= 0.5 and Pr = 6.2**.

**Figure 7:**
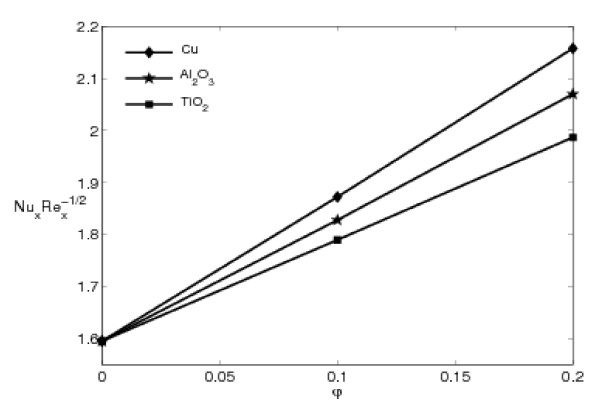
**Variation of the local Nusselt number NuxRex-1/2 with *φ *for different nanoparticles with *ε *= 0.5 and Pr = 6.2**.

Figures 6 and 7 illustrate the variations of the skin friction coefficient and the local Nusselt number, given by Eqs. 13 and 14 with the nanoparticle volume fraction parameter *φ *for three different of nanoparticles: copper (Cu), alumina (Al_2_O_3_), and titania (TiO_2_) with *ε *= 0.5. These figures show that these quantities increase almost linearly with *φ*. The presence of the nanoparticles in the fluids increases appreciably the effective thermal conductivity of the fluid and consequently enhances the heat transfer characteristics, as seen in Figure 7. Nanofluids have a distinctive characteristic, which is quite different from those of traditional solid-liquid mixtures in which millimeter- and/or micrometer-sized particles are involved. Such particles can clot equipment and can increase pressure drop due to settling effects. Moreover, they settle rapidly, creating substantial additional pressure drop [41]. In addition, it is noted that the lowest heat transfer rate is obtained for the TiO_2 _nanoparticles due to domination of conduction mode of heat transfer. This is because TiO_2 _has the lowest thermal conductivity compared to Cu and Al_2_O_3_, as presented in Table 1. This behavior of the local Nusselt number is similar with that reported by Oztop and Abu-Nada [39]. However, the difference in the values for Cu and Al_2_O_3 _is negligible. The thermal conductivity of Al_2_O_3 _is approximately one tenth of Cu, as given in Table 1. However, a unique property of Al_2_O_3 _is its low thermal diffusivity. The reduced value of thermal diffusivity leads to higher temperature gradients and, therefore, higher enhancement in heat transfers. The Cu nanoparticles have high values of thermal diffusivity and, therefore, this reduces the temperature gradients which will affect the performance of Cu nanoparticles.

**Figure 8:**
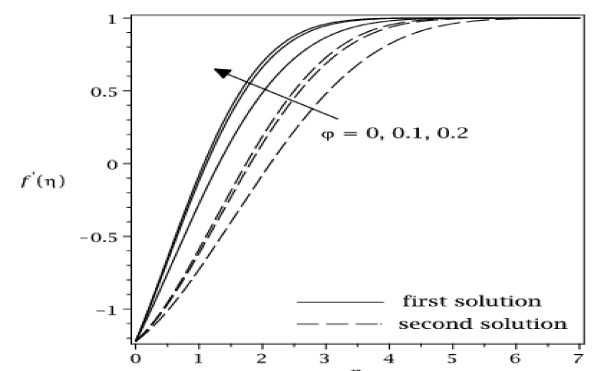
**Velocity profiles for some values of *φ *(0 ≤ *φ *≤ 0.2) for Cu-water working fluid with *ε *= -1.22 and Pr = 6.2**.

**Figure 9:**
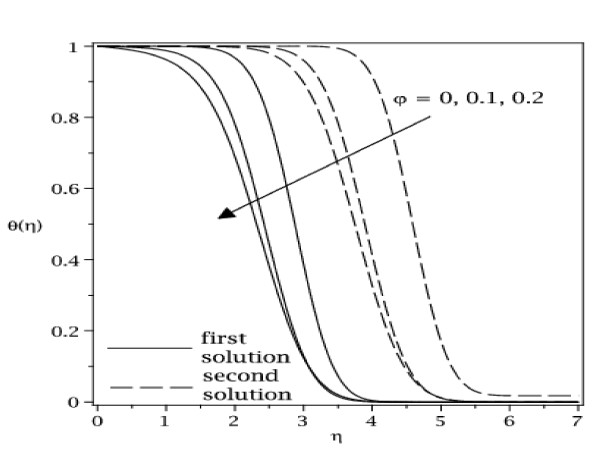
**Temperature profiles for some values of *φ *(0 ≤ *φ *≤ 0.2)for Cu-water working fluid with *ε *= -1.22 and Pr = 6.2**.

**Figure 10:**
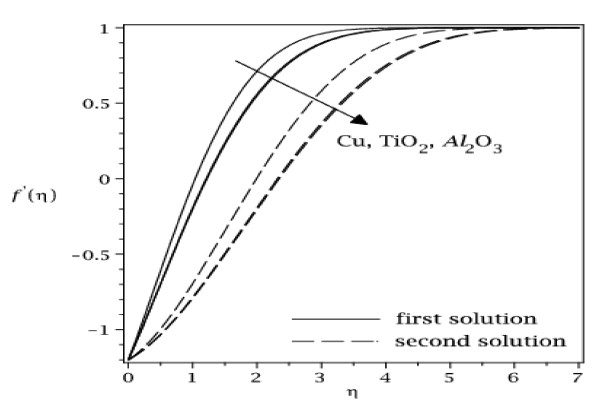
**Velocity profiles for different nanoparticles with *φ *= 0.1, *ε *= -1.2 and Pr = 6.2**.

**Figure 11:**
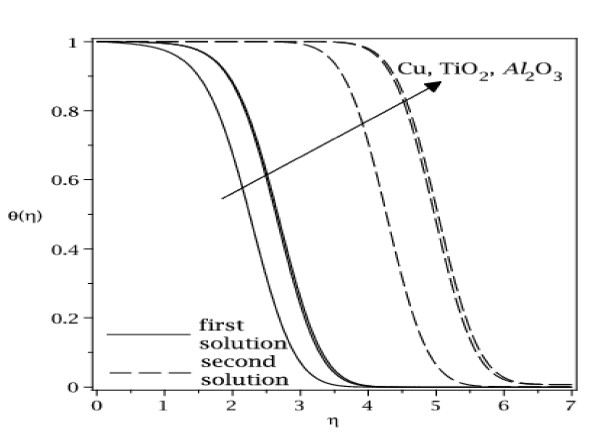
**Temperature profiles for different nanoparticles with *φ *= 0.1, *ε *= -1.2 and Pr = 6.2**.

The samples of velocity and temperature profiles for some values of parameters are presented in Figures 8, 9, 10, and 11. These profiles have essentially the same form as in the case of regular fluid (*φ *= 0). The terms first solution and second solution refer to the curves shown in Figures 2, 3, 4, and 5, where the first solution has larger values of *f*"(0) and -*θ'*(0) compared to the second solution. Figures 8, 9, 10, and 11 show that the far field boundary conditions (9) are satisfied asymptotically, thus support the validity of the numerical results, besides supporting the existence of the dual solutions shown in Table 2 as well as Figures 2, 3, 4, and 5.

## Conclusions

We have presented an analysis for the flow and heat transfer characteristics of a nanofluid over a stretching/shrinking sheet in its own plane. The stretching/shrinking velocity and the ambient fluid velocity are assumed to vary linearly with the distance from the stagnation point. The resulting system of nonlinear ordinary differential equations is solved numerically for three types of nanoparticles, namely copper (Cu), alumina (Al_2_O_3_), and titania (TiO_2_) in the water-based fluid with Prandtl number Pr = 6.2, to investigate the effect of the solid volume fraction parameter *φ *on the fluid and heat transfer characteristics. Different from a stretching sheet, it is found that the solutions for a shrinking sheet are non-unique. The inclusion of nanoparticles into the base water fluid has produced an increase in the skin friction and heat transfer coefficients, which increases appreciably with an increase of the nanoparticle volume fraction. Nanofluids are capable to change the velocity and temperature profile in the boundary layer. The type of nanofluids is a key factor for heat transfer enhancement. The highest values of the skin friction coefficient and the local Nusselt number were obtained for the Cu nanoparticles compared with the others.

## Competing interests

The authors declare that they have no competing interests.

## Authors' contributions

NB and AI performed the numerical analysis and wrote the manuscript. IP carried out the literature review and co-wrote the manuscript. All authors read and approved the final manuscript.
